# Identifying targets for antibiotic stewardship interventions in pediatric patients in Punjab, Pakistan: point prevalence surveys using AWaRe guidance

**DOI:** 10.3389/fped.2024.1469766

**Published:** 2025-01-10

**Authors:** Samia Sheikh, Zikria Saleem, Shairyar Afzal, Muhammad Usman Qamar, Ali Abuzar Raza, Syed Zeeshan Haider Naqvi, Mahmood Basil A. Al-Rawi, Brian Godman

**Affiliations:** ^1^ Department of Pharmacy Practice, Faculty of Pharmacy, Bahauddin Zakariya University, Multan, Pakistan; ^2^ Department of Pharmacy, DHQ Hospital Jhelum, Jhelum, Pakistan; ^3^ Institute of Microbiology, Faculty of Life Sciences, Government College University Faisalabad, Faisalabad, Pakistan; ^4^ Division of Infectious Diseases, Department of Medicine, Geneva University Hospitals and Medical School, Geneva, Switzerland; ^5^ Institute of Molecular Biology and Biotechnology (IMBB), The University of Lahore, Lahore, Pakistan; ^6^ Department of Microbiology, CMH Multan Institute of Medical Sciences, Multan, Pakistan; ^7^ Department of Optometry, College of Applied Medical Sciences, King Saud University, Riyadh, Saudi Arabia; ^8^ Department of Public Health Pharmacy and Management, School of Pharmacy, Sefako Makgatho Health Sciences University, Ga-Rankuwa, South Africa; ^9^ Strathclyde Institute of Pharmacy and Biomedical Sciences, University of Strathclyde, Glasgow, United Kingdom

**Keywords:** pediatric, antimicrobial resistance (AMR), antimicrobial stewardship (AMS), Pakistan, point prevalence survey, WHO AWaRe

## Abstract

**Introduction:**

Surveillance of antibiotic use is crucial for identifying targets for antibiotic stewardship programs (ASPs), particularly in pediatric populations within countries like Pakistan, where antimicrobial resistance (AMR) is escalating. This point prevalence survey (PPS) seeks to assess the patterns of antibiotic use in pediatric patients across Punjab, Pakistan, employing the WHO AWaRe classification to pinpoint targets for intervention and encourage rational antibiotic usage.

**Methods:**

A PPS was conducted across 23 pediatric wards of 14 hospitals in the Punjab Province of Pakistan using the standardized Global-PPS methodology developed by the University of Antwerp. The study included all pediatric inpatients receiving antibiotics at the time of the survey, categorizing antibiotic prescriptions according to the WHO Anatomical Therapeutic Chemical classification and the AWaRe classification system.

**Results:**

Out of 498 pediatric patients, 409 were receiving antibiotics, representing an antibiotic use prevalence of 82.1%. A substantial majority (72.1%) of the prescribed antibiotics fell under the WHO's Watch category, with 25.7% in the Access category and 2.2% in the Reserve group. The predominant diagnoses were respiratory infections, notably pneumonia (32.4%). The most commonly used antibiotics were ceftriaxone (37.2%) and Vancomycin (13.5%). Only 2% of antibiotic uses were supported by culture sensitivity reports, highlighting a reliance on empirical therapy.

**Conclusion:**

The high prevalence of antibiotic use, particularly from the Watch category, and low adherence to culture-based prescriptions underscore the critical need for robust antibiotic stewardship programs in Pakistan. Strengthening these programs could help mitigate AMR and optimize antibiotic use, aligning with global health objectives.

## Introduction

Increasing antimicrobial resistance (AMR) hastens the arrival of the post-antibiotic era, which is a concern necessitating a range of different activities (1, 2). Extensive misuse of antibiotics contributes to increasing AMR, potentially reversing decades of medical accomplishments (3, 4). In 2021, it was estimated globally, there were 1.14 million deaths due to AMR, with 1 in 5 deaths occurring in children under 5 years of age, with the highest rates of AMR in low- and middle-income countries (LMICs) (5, 6). This is set to rise to 8.22 million deaths by 2050 alongside considerable morbidity, combined with an annual economic loss of approximately $US1 trillion unless addressed (6–8).

Antibiotics are the most commonly prescribed medicines in the pediatric population in both community and hospital settings with potentially inappropriate usage, with consumption increasing 46% between 2000 and 2018 in children under 5 years of age (9, 10). Antibiotic consumption also appreciably increased among sick children under the age of 5 in LMICs between 2005 and 2017, greatest in South-East Asia (11). This is a concern increasing both AMR as well as adverse events (12), with rapidly increasing resistant organisms, including gram-negative bacilli and *Staphylococcus aureus* (13). The pediatric population is particularly vulnerable to the consequences of AMR, resulting in increasing morbidity and mortality (12, 14, 15). Addressing this is a critical issue to reach the agreed Sustainable Development Goal to reduce newborn mortality, with Pakistan a key country with high mortality rates compared to a number of other LMICs (16, 17). Moreover, limitations pertaining to eligibility to participate in clinical trials impact optimal data generation and development, potentially enhancing AMR (18).

Concerns with growing rates of AMR and associated consequences have resulted in the instigation of the Global Action Plan (GAP) by the World Health Organization (WHO) to tackle AMR, leading to the development of National Action Plans (NAPs) emanating from the GAP also mimic objectives laid out in the latter (19, 20). However, there have been concerns with the implementation of NAPs, including both personnel and resource issues, as seen in Pakistan and other LMICs (21–23). The WHO also developed the Global Antimicrobial Resistance and Use Surveillance (GLASS) in 2017, given the considerable need to track resistance development (24). However, there are considerable challenges with the current report accentuating the limited surveillance capacities of LMICs to record resistance and antibiotic use data (25, 26). The WHO also introduced the AWaRe classification system in 2017, categorizing antibiotics into Access, Watch and Reserve classes (27). The AWaRe framework intends to work as a means of surveillance and act as a stewardship tool (28, 29). Alongside this, the WHO developed its own Point Prevalence Survey (PPS) methodology to document antibiotic use in hospitals, building on Global and ECDC methodologies (30–33). Antibiotic stewardship programs (ASPs) are increasingly seen as an effective means to enhance the appropriate use of antibiotics and reduce AMR and costs (34–36). However, there can be concerns among LMICs to implement ASP due to human resource shortages, diagnostic inadequacies, poor antibiotic use monitoring and surveillance capacity, as well as personnel shortages (37–40). The WHO has also recently provided guidance on undertaking ASPs (41). ASP implementation is particularly important in promoting judicious antibiotic use in the pediatric population, given current challenges (10, 42–44).

Although the NAP Pakistan against AMR has been in effect since 2017, Pakistan acknowledges AMR as a potential threat, whereas the ground realities of the AMR situation are dissent from what was pledged in official documents (23, 45–47) and inadequacies are conspicuous in implementing ASP protocols (48, 49). Pakistan is struggling in the battle against growing bacterial resistance (50–52). There have also been reports of a high prevalence of multi-drug resistance organisms (MDRO) among children in pediatric wards in Pakistan (53). Consequently, there is a need to urgently document current antibiotic utilization patterns among neonates and children in hospitals across Punjab, Pakistan. We are aware of published papers documenting concerns with high use of antibiotics, including high use of “Watch” antibiotics with their greater resistance potential, among the pediatric population in a limited number of hospitals in Pakistan, including tertiary hospitals (54–57). However, there is a need to update this, including evidence from a greater number of hospitals, to inform future ASPs where concerns have been identified. Consequently, the current study intends to expand on previous PPS studies in the pediatric population using the WHO AWaRe classification.

## Methods

### Study design and setting

This point prevalence survey (PPS) was conducted using the standardized Global Point Prevalence Survey on Antimicrobial Consumption and Resistance (Global-PPS) methodology developed by the University of Antwerp. The Anatomical Therapeutic Chemical (ATC) and AWaRe classification system of the WHO were employed to classify the different antibiotics used. Data were collected from 23 wards across 14 hospitals in Punjab, the most populous province of Pakistan, known for its diverse healthcare challenges and significant role in national health outcomes. The hospitals surveyed included a mix of seven secondary and six tertiary care facilities, one specialized cancer hospital, and a combination of ten public and four private sector hospitals. Participation in the survey was voluntary, ensuring that only those institutions willing to engage in this study were included, as mentioned in the Global PPS method.

### Sampling and inclusion criteria

Pediatric patients (0–18 years) admitted to medical and surgical wards, pediatric intensive care units, and neonatal units at 8:00 a.m. on the day of the survey were included. Patients receiving at least one systemic antibiotic at the time of data collection formed the numerator, while all admitted pediatric patients on the surveyed wards comprised the denominator.

### Data collection tools

Data collection was conducted using the standardized Global-PPS ward and patient forms. At the ward level, data included the total number of admitted patients, the total number of beds, and type of ward. At the patient level, detailed information was collected on demographics (age and gender), diagnosis and type of infection (e.g., community-acquired or hospital-acquired), the indication for antibiotic use (therapeutic or prophylactic), and the antimicrobial agent(s) prescribed, including the dose, route, and frequency of administration.

### Data collection procedure

All pediatric inpatients receiving antimicrobial therapy at 08:00 a.m. were included, and data collection forms were completed for these patients only. All prescribed antimicrobials at the time of the survey were recorded. Patients who were transferred to another ward after 08:00 a.m. were included as part of the initial ward of admittance. Neonates born before 08:00 a.m. on the day of the survey were included. The last prescribed antibiotic was recorded if the prophylaxis or treatment was changed on the day of the survey before or at 08:00 a.m. Surgical wards were not surveyed on a day following a holiday but on other weekdays to capture information about prophylaxis in the last 24 h. Surgical prophylaxis included agents to prevent surgical site infections, and long-acting antibiotics or intermittent treatments given within 24 h before the survey were included. Medical prophylaxis was defined as the use of antibiotics to prevent infections in patients with specific medical conditions. Infections were classified as community-acquired infections (CAIs) if symptoms started less than 48 h from admission to the hospital or were present on admission. Hospital-acquired infections (HAIs) were defined as infections with symptoms starting 48 h after admission. The data collection was conducted by using the Global PPS standardized methodology to ensure consistency and adherence to standards. All available information documented in the Global PPS form was thoroughly collected. The detailed method is described on the Global-PPS website (58). Information included patients' demographics, diagnosis, infection type and prescribed antibiotics details. Data was collected from patients' medical notes and prescribing charts. Additional details from patients' medical case notes were recorded after discussions with nursing staff and physicians, when necessary, especially if crucial data such as antibiotic selection was missing. However, in most cases, only patients' notes were reviewed. The collected data were double-checked for completeness and accuracy to rule out any missing or inconsistent information. There was no direct contact with any patient during the data collection process in line with other PPS studies. Data from each ward being completely surveyed within 1 day to minimize the effect of patient movement between wards and within the hospital. The data was collected in March 2024. Patients' anonymity was maintained throughout the process.

### Statistical analysis

Data were exported from the Global-PPS web application into a Microsoft Excel database for analysis. Statistical analyses were performed using SPSS version 25 (IBM Corporation, Armonk, NY, USA). In this study, we utilized both descriptive and inferential statistical methods to analyze the patterns of antibiotic usage across the AWaRe classifications (Access, Watch, Reserve) under varied clinical settings. Initially, descriptive statistics provided a foundational understanding of the data distribution and characteristics. Further, cross-tabulations were employed to examine the distribution and usage patterns of antibiotics classified as Access, Watch, and Reserve, highlighting the frequency and context of their utilization across different patient groups and departments. Subsequent to the descriptive analysis, logistic regression was applied to more deeply investigate the factors influencing the likelihood of antibiotics being classified under the “Watch” category. This method facilitated the quantification of the impact of various factors, including department type, patient age group, and diagnosis, on the AWaRe classification, allowing for a critical analysis of trends and associations within the data. The “Access” vs. “Watch” ratio was also calculated to assess the relative use given concerns with the increasing use of “Watch” antibiotics in LMICs in recent years, facilitating the identification of targets for stewardship interventions (59). The United Nations General Assembly (UNGA), in its meeting in September 2024, has set a global target of “Access” antibiotics to account for 70% of total utilization to counter AMR (60).

### Ethical considerations

Ethical clearance for the study was obtained from the Human Ethics Division of the Department of Pharmacy Practice at the Faculty of Pharmacy, Bahauddin Zakariya University, Multan (Ref: BZU-FOPDPP-2446). Permission to conduct the study within the selected hospitals was also granted by their respective management teams. To ensure confidentiality, all patient data were anonymized at the time of collection. Unique, non-identifiable survey numbers generated by the Global-PPS software were used to maintain the anonymity of the data.

## Results

The PPS was conducted across 23 pediatric wards of 14 hospitals, encompassing a total of 692 beds. Out of 498 pediatric patients assessed, 409 patients were prescribed antibiotics, giving a prevalence of 82.1%. The survey recorded a total of 734 instances where antibiotics were used across these pediatric cases. Around 327 patients were using more than one antibiotic. Only 2% of antibiotic use was based on culture sensitivity reports, and in only 23.2% of cases was the reason for prescribing an antibiotic mentioned on the patient notes. “Watch” antibiotics accounted for 72.1% (529) of the 734 antibiotics prescribed, followed by 25.7% of antibiotics in the “Access” category (Table 1). The highest prescribing of antibiotics was in the pediatric medical wards at 83.5%, with the distribution of antibiotics based on the AWaRe categories closely mirrored the overall findings. The Intensive Care Unit, although smaller in scale, showed a higher proportion of Reserve category antibiotics at 5.9% for central nervous system infections and prophylaxis, suggesting a focused approach to managing severe or high-risk infections. Parenteral administration dominated the route of administration, comprising 96.6% of the prescriptions.

**Table 1:** Distribution of antibiotic use by AWaRe classification.

Variables	Access		Watch		Reserve		Total	
	*n*	%	*N*	%	*n*	%	*n*	%
AWaRe	189	25.7	529	72.1	16	2.2	734	100
Department type								
Pediatric medical ward	159	25.9	439	71.6	15	2.4	613	83.5
Hematology-oncology PMW	8	15.1	45	84.9	0	0.0	53	7.2
Neonatal medical ward	11	21.6	40	78.4	0	0.0	51	6.9
Intensive care unit	11	64.7	5	29.4	1	5.9	17	2.3
Sex								
Male	113	25.2	326	72.6	10	2.2	449	61.2
Female	76	26.7	203	71.2	6	2.1	285	38.8
Age group								
Neonates (1–30 days)	22	29.7	51	68.9	1	1.4	74	10.1
Infant (1–12 months)	94	27.9	235	69.7	8	2.4	337	45.9
Pediatrics (Above 1 year)	73	22.6	243	75.2	7	2.2	323	44.0
Route AWaRe								
Parenteral	183	25.8	510	71.9	16	2.3	709	96.6
Oral	6	24.0	19	76.0	0	0.0	25	3.4
Diagnosis								
Pneumonia	75	31.5	158	66.4	5	2.1	238	32.4
Central nervous system infection	18	14.8	99	81.1	5	4.1	122	16.6
Sepsis	30	35.3	54	63.5	1	1.2	85	11.6
Gastrointestinal infection	11	15.9	57	82.6	1	1.4	69	9.4
Prophylaxis for respiratory pathogens	10	27.0	27	73.0	0	0.0	37	5.0
Newborn medical prophylaxis	5	17.2	24	82.8	0	0.0	29	4.0
Medical prophylaxis in general	5	17.9	23	82.1	0	0.0	28	3.8
Neutropenic patient fever	5	22.7	17	77.3	0	0.0	22	3.0
Prophylaxis for gastrointestinal tract	6	35.3	10	58.8	1	5.9	17	2.3
Upper respiratory tract infection	5	38.5	8	61.5	0	0.0	13	1.8
Pyrexia of unknown origin	4	36.4	7	63.6	0	0.0	11	1.5
Others	15	23.8	45	71.4	3	4.8	63	8.6
Indication								
Community acquired infections	154	26.0	423	71.3	16	2.7	593	80.8
Medical prophylaxis	28	25.0	84	75.0	0	0.0	112	15.3
Healthcare associated infections	6	31.6	17	68.4	0	0.0	23	3.1
UNK	0	0.0	4	100.0	0	0.0	4	0.5
Surgical prophylaxis	1	50.0	1	50.0	0	0.0	2	0.3
Reason in notes	55	32.4	115	67.6	0	0.0	170	23.2

**Table 2:** Commonly prescribed antibiotics.

Antibiotic name	ATC code	AWaRe class	*n*	%
Ceftriaxone	J01DD04	Watch	273	37.2
Vancomycin	J01XA01	Watch	99	13.5
Ampicillin	J01CA01	Access	58	7.9
Amikacin	J01GB06	Access	53	7.2
Meropenem	J01DH02	Watch	44	5.9
Amoxicillin and enzyme inhibitor	J01CR02	Access	31	4.2
Piperacillin and enzyme inhibitor	J01CR05	Watch	31	4.2
Metronidazole	J01XD01	Access	22	3.0
Cefotaxime	J01DD01	Watch	21	2.9
Benzylpenicillin	J01CE01	Access	17	2.3
Linezolid	J01XX08	Reserve	14	1.9
Ciprofloxacin	J01MA02	Watch	12	1.6
Ceftazidime	J01DD02	Watch	10	1.4
Azithromycin	J01FA10	Watch	9	1.2
Cefuroxime	J01DC02	Watch	6	0.8
Clarithromycin	J01FA09	Watch	6	0.8
Gentamicin	D06AX07	Access	4	0.5
Ofloxacin	S01AE01	Watch	4	0.5
Sulfamethoxazole and trimethoprim	J01EE01	Access	4	0.5
Levofloxacin	J01MA12	Watch	4	0.5
Others	-	-	12	1.6

**Table 3:** Factors influencing the use of “watch” category antibiotics.

Variable	Coefficients	Odds ratios	*p*-value	95% CI lower	95% CI upper
Constant	1.96	7.12	0.00	0.72	3.21
Gender male	0.16	1.18	0.46	−0.27	0.59
Department type					
Intensive care unit	−3.90	0.02	0.01	−6.79	−1.00
Neonatal medical ward	−1.89	0.15	0.14	−4.39	0.61
Paediatric medical ward	−0.53	0.59	0.31	−1.56	0.50
Age group					
Neonate	1.12	3.07	0.34	−1.20	3.44
Ped age above 1 year	0.14	1.16	0.53	−0.30	0.59
Diagnosis site					
GI	−0.41	0.67	0.34	−1.25	0.44
NDS	−0.70	0.50	0.07	−1.47	0.06
Neonatal	−0.18	0.84	0.82	−1.69	1.33
RESP	−0.83	0.44	0.02	−1.51	−0.16
Indication					
HAI	0.65	1.91	0.43	−0.96	2.25
MP	0.39	1.48	0.29	−0.33	1.11

The most frequently reported diagnosis was pneumonia (32.4%), predominantly treated with Watch group antibiotics. Among the individual antibiotics, ceftriaxone, a “Watch” antibiotic, was the most commonly prescribed antibiotic, accounting for 37.2% of all antibiotics used, categorized under the Watch group, followed by vancomycin (13.5%) (Table 2). In examining the determinants influencing the use of “Watch” antibiotics, logistic regression analysis revealed several significant factors. The intercept, or constant term, of the model, was positive (coefficient = 1.96, *p*-value = 0.00), indicating the baseline log-odds of an antibiotic being classified as “Watch” when all other variables are zero. This result suggests a baseline propensity towards the “Watch” classification in the absence of modifying factors. Significantly, antibiotics administered within the ICU were substantially less likely to be classified as “Watch,” with a coefficient of −3.89 (*p*-value = 0.01) and an odds ratio of 0.02. This indicates a 98% decrease in the odds of “Watch” classification for antibiotics used in the ICU compared to the reference department, underscoring a conservative approach to antibiotic use in this high-risk setting (Table 3).

## Discussion

Unequivocally, topnotch surveillance of antibiotic use is of prime importance to inform stewardship interventions, in which the PPS methodology offers a preeminent surveillance potential to monitor the use of antibiotics and acts as a baseline to inform policies regarding the correct utilization of antibiotics in hospital settings (11, 30). We believe this is the first comprehensive study undertaken in Pakistan to assess current antibiotic utilization rates among pediatric patients along with providing a comprehensive breakdown of antibiotic use across indications, wards and age groups. The prevalence rate of 82.1% for antibiotic use is similar to previous studies in Pakistan, including Mustafa et al. at 95.5% (56) and 97.5% (55) as well as Ambreen et al., with rates varying between 91% for the pediatric medical ward to 99% for pediatric intensive care and the neonatal medical ward (57). In addition, similar to a study in South Africa with a prevalence rate of 92% among hospitalized children (61) and in India, where up to 89% of neonates in NICUs were prescribed antibiotics (62). However, lower than seen in China (56.8%–66.1%) (63, 64), another study in India (61.5%) (65), Jordan at 75.6% overall, although up to 82.2% in Pediatric wards (66), Myanmar at 63.4% (67) and another study in South Africa at 49.7% (68). This compares with high-income countries that have shown decreasing trends in antibiotic utilization rates potentially enhanced by established ASPs, as well as adequate diagnostic and monitoring facilities compared to LMICs (69), where there is a dire need to upgrade existing laboratory infrastructure coupled with improving quality standards through international accreditation and standardizing procedures (70). The lack of monitoring of patient records as part of ASPs may help explain the fact that in only 23.2% of instances in our study, it was the reason for prescribing antibiotics mentioned in patients' notes. This compares to a Belgian study in which, on 81.9% of occasions, the indication for antibiotic use was recorded (33).

The high prevalence if antibiotic use may also have been exacerbated by the fact that in only 2% of occasions was culture and sensitivity testing undertaken. Whilst this is similar to other studies in Pakistan due to financial constraints (54, 56, 71), this is a concern going forward that needs to be urgently addressed along with addressing the lack of antibiograms to guide prescribing building on treatment recommendations in the recently launched AWaRe book (40, 72–74).

Our study findings revealed that pneumonia was the most prevalent infection for which antibiotics were prescribed (32.4%), similar to other studies from Pakistan (54–56). Given the epidemiological pattern, current diagnostic procedures may also need to be standardized as well because of the unreliability issues with the typically opted procedure of respiratory rate count in LMICs (75). An Ethiopian study also identified pneumonia as the most prevalent underlying infectious disease (28.6%) requiring antibiotics (76). However, a Jordanian study reported a lower prevalence of pneumonia at 20.6% among the underlying clinical conditions for the prescribing of antibiotics (66). Surprisingly, healthcare-associated infections (HAIs) were only noted in 3.1% of the patients as compared to 80.8% for community-acquired infections (CAIs). However, similar to the findings of Mustafa et al. (2022) (56). This result could be due to a lower reporting rate of HAIs in our study due to limited diagnostic modalities available, with Arif et al. (2021) reporting a higher rate at 20.3% (54). HAIs need to be addressed by strengthening the implementation of infection prevention and control programs (IPC) within hospital settings where this is an issue due to concerns about developing infections with multi-drug resistant organisms (MDROs). Hospitals may also benefit from the synergistic effect of combining infection prevention and control programs (IPCs) and ASPs to minimize resistant infections and antibiotic use (77, 78). This will be monitored in the future.

Parenteral administration was high at 96.6%, which is similar to other studies undertaken: Brazil (91%) (79), Pakistan (91.5%) (80), Ethiopia (90.5%) (76) and India (77.9%) (81). This also needs to be looked at in the future as part of possible ASPs with parenteral formulations and routes associated with an increased risk of infection (catheter-related infection) and higher costs if IV-to-oral switching is delayed (82). Increasing evidence suggests that an IV-to-Oral switching where this is practical is associated with improved clinical and economic outcomes (83, 84).

Another key concern is the high consumption of “Watch” category antibiotics in our study at 72.1%, indicating an appreciable divergence from the recent UNGA target of up to 70% consumption of “Access” antibiotics (60). However, such a non-judicious antibiotic use could be impeded by preferring ASP interventions, particularly formulary restriction or prior authorization of antibiotics (85, 86). Our findings are consistent with multifarious studies reporting inappropriate and high Watch category usage in various countries and worldwide (59, 64, 87–90). In contrast, South African studies (68, 91) reported 55.9%–70.2% antibiotic prescriptions from the Access category. Ceftriaxone was the most commonly used antibiotic in our study (37.2%), which is similar to other studies in Pakistan (25.8%) (56) as well as Ethiopia (30.4%) (76), and Uganda (50.6%) (92). Moreover, a point prevalence survey in 69 countries by Pauwels et al. concluded that Ceftriaxone is the most prescribed drug globally, predominantly used against pneumonia, with total prescriptions accounting for 20% (87).

Overuse of ceftriaxone has substantially contributed to its growing resistance against gram-negative pathogens (*Klebsiella pneumoniae and E. Coli*) in the pediatric population (50), which needs to be addressed going forward. Recently, a pharmacist-led educational intervention in one of the secondary care hospital in Pakistan showed an improvement in the knowledge, attitude, and practices of healthcare workers regarding the rational use of antibiotics and concluded that continuous educational programs could foster strong adherence to ASP guidelines (93). Such programs should be reciprocated to other facilities to generate strong compliance with ASP and curtail the threat of growing AMR.

The present study is not absolved of limitations. Although the study is multi-centric, we prefer not to generalize the results to Pakistan, as repeated PPS from all the provinces is a more balanced approach towards identifying trends affecting prescribing determinants. The study may not fully capture the influence of seasonal fluctuations on different diseases and use of antibiotics. The present study noted fewer antibiotic prescriptions in surgical prophylaxis as the survey day was “surgical OT day”; most of the patients were present in the operating room. Moreover, owing to the minimal reporting of antibiotic indications on patient notes, it was not possible to judge the appropriateness of the prescribed antibiotic. However, in conjunction with the existing literature, our study provides useful information regarding the state of antibiotic use in pediatrics and identifies areas of improvement and ASP implementation. Based on the findings, healthcare facilities can enhance guideline adherence and rationalize prescribing practices, contributing to better patient outcomes and reduced AMR in resource-limited settings.

## Conclusion

This study reported a high prevalence of antibiotic use in pediatric patients. The use of the Watch category antibiotics was high, presenting a divergence from the target set by the WHO's AWaRe Classification. Although the AWaRe framework acts as a tool to instigate ASP, the practice of antibiotic use noted in our study highlights a clear deviation. Strong measures should be taken to ensure adherence to this tool. Moreover, the high empirical use of antibiotics highlights the lack of a microbiological laboratory infrastructure. It is time for ASP to be strengthened within hospital facilities to curb rising AMR and protect antibiotics for future use.

## Data Availability

The raw data supporting the conclusions of this article will be made available by the authors, without undue reservation.
